# Proteomics Landscape of Alzheimer’s Disease

**DOI:** 10.3390/proteomes9010013

**Published:** 2021-03-10

**Authors:** Ankit P. Jain, Gajanan Sathe

**Affiliations:** 1 Institute of Bioinformatics, International Technology Park, Bangalore 560066, India; ankit@ibioinformatics.org; 2 Manipal Academy of Higher Education (MAHE), Manipal 576104, India

**Keywords:** Alzheimer, mass spectrometry, proteomics, targeted proteomics

## Abstract

Alzheimer’s disease (AD) is the most prevalent form of dementia, and the numbers of AD patients are expected to increase as human life expectancy improves. Deposition of β-amyloid protein (Aβ) in the extracellular matrix and intracellular neurofibrillary tangles are molecular hallmarks of the disease. Since the precise pathophysiology of AD has not been elucidated yet, effective treatment is not available. Thus, understanding the disease pathology, as well as identification and development of valid biomarkers, is imperative for early diagnosis as well as for monitoring disease progression and therapeutic responses. Keeping this goal in mind several studies using quantitative proteomics platform have been carried out on both clinical specimens including the brain, cerebrospinal fluid (CSF), plasma and on animal models of AD. In this review, we summarize the mass spectrometry (MS)-based proteomics studies on AD and discuss the discovery as well as validation stages in brief to identify candidate biomarkers.

## 1. Introduction

Alzheimer’s disease (AD) is the most common progressive neurodegenerative disorder with memory loss, cognitive impairment, disorientation, and psychiatric symptoms. These characteristics of AD render patients incapable of performing day-to-day activities independently. With an annual global increase in patients with cognitive impairment, the cost of caring for these patients is increasing. As the number of AD-affected people increases due to the aging population, it is becoming one of the greatest healthcare challenges of the 21st century [1]. The major hallmark of AD is extracellular deposition of amyloid-β (Aβ) as plaques in the brain and the formation of intracellular neurofibrillary tangles (NFTs) composed of the accumulation of hyperphosphorylated tau (p-tau). These two events are the major cause of neuronal death [2,3]. Despite advancements in research, definitive causes of the disease remain elusive with no effective treatments available yet [4]. Existing therapies for AD do not prevent the progression of the disease, and several drugs have failed in recent trials. As the understanding of AD pathogenesis is limited, it is difficult to develop novel therapies for AD. Therefore, further understanding of molecular mechanisms and cellular signaling pathways that are responsible for AD pathogenesis is needed for discovering new targets and evolving treatment strategies for AD. Mass-spectrometry-based proteomics will continue to play an important role in the future for discovering these events responsible for AD pathogenesis.

The current clinical diagnosis of AD heavily relies on neuropsychological testing, clinical assessment, imaging, and exclusion of other neurological disorders [5]. Presently, amyloid-beta (Aβ) and tau proteins in CSF are the only molecular markers for AD diagnosis used in the clinics. Both, the increase in Tau protein levels as well as its hyper-phosphorylation in the cerebrospinal fluid (CSF) along with lower Aβ1–42 (Aβ42) has shown higher sensitivity and specificity in discriminating AD from controls [6,7]. Although these markers are used for clinical diagnosis, the major limitations are early detection of the disease, differential diagnosis of various dementias, and monitoring of advancement in disease and response to therapy [8]. Thus, there is an unmet need for the discovery of new biomarkers as well as rigorous validation of current markers on larger and independent cohorts. Next-generation proteomics technologies such as targeted proteomics and data independent acquisition-based proteomics hugely augment such efforts.

In this review, we will describe the different biological samples and proteomics technologies that have been used for furthering our understanding of disease pathogenesis and the discovery of candidate biomarkers.

## 2. Proteomics Studies in AD Pathogenesis and Biomarker Discovery

**Figure 1:**
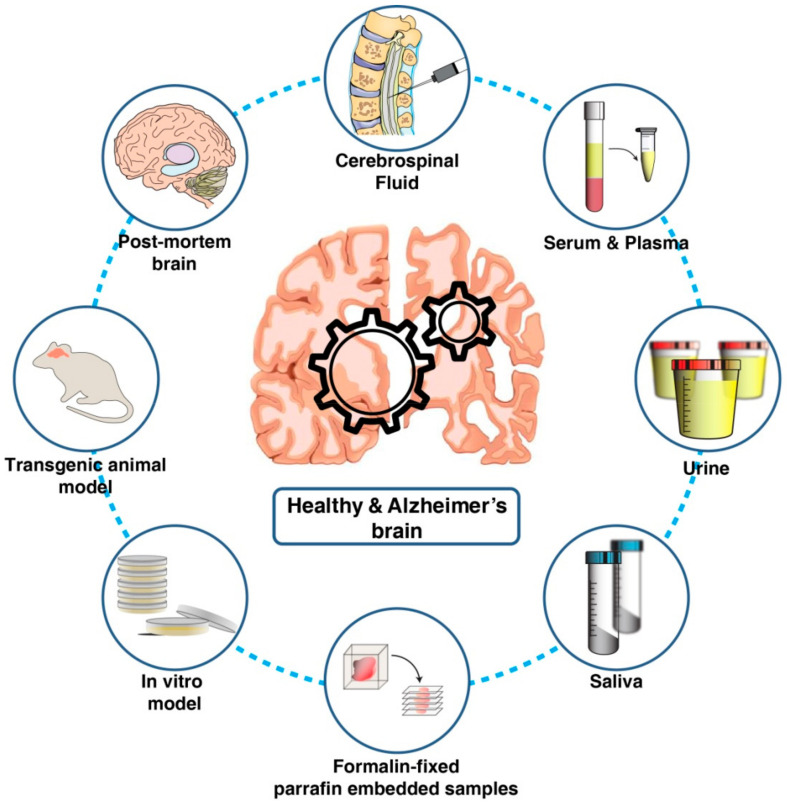
Biological materials used for understanding Alzheimer’s pathology and for the discovery and validation of candidate biomarkers using proteomics technologies.

Over the past few decades, several studies on both patient-derived biological samples as well as in vivo and in vitro Alzheimer’s disease models utilizing proteomics technologies have been carried out. These studies have contributed greatly toward the detection of a biomarker as well as understanding the disease pathogenesis (Figure 1).

### 2.1. Proteomics and AD Pathogenesis

#### 2.1.1. Human Brain Tissue Samples

Understanding the function of the brain and the related disease mechanisms requires comprehensive knowledge of the changes in the neuronal proteome and post-translational modifications (PTMs). The past decade has seen remarkable advances in high-resolution mass-spectrometry-based proteomics that have enabled precise analysis of thousands of proteins from brain tissue samples in a relatively shorter amount of time. Quantitative proteome analysis of the human hippocampus at different Braak stages identified alteration in 372 proteins. These proteins were components of the extracellular matrix and calcium-dependent signaling [9]. Similarly, Munsunuri et al. used dimethyl labeling to compare proteome changes in temporal neocortex in Alzheimer’s disease (AD) patients and non-AD individuals. They observed significant alterations in 69 proteins involved in several pathways including energy metabolism, glycolysis, oxidative stress, apoptosis, signal transduction, and synaptic functioning [10]. Furthermore, label-free quantitative proteomics of frontal tissues from AD and control brain samples by Zhang et al. demonstrated the involvement of diverse signaling networks including proteostasis, RNA homeostasis, neuroinflammation, lipid homeostasis, and myelin-axon interactions among others to be associated with Alzheimer’s pathology [11]. Eric et al. measured 6533 proteins from dorsolateral prefrontal cortex across AD, AsymAD, and controls using an isobaric tandem mass tag (TMT)-based quantitative approach. They identified changes in several novel proteins involved in mRNA splicing and also observed a correlation of these proteins with known markers of AD pathology and cognitive decline [12]. Our group has also carried out an in-depth analysis of the frontal gyrus of the AD patients and age-matched control individual. This led to the identification of 8066 proteins and alteration of 432 proteins in the AD brain [13]. Apart from these, there are several large-scale analyses of brain proteomes of AD individuals that have identified alterations in energy metabolism, amyloidosis, immune response, mitochondrial, and synaptic functions. These studies identified more than 10,000 proteins on large number of the samples [14,15,16,17,18]. Overall, these studies show that a gamut of cellular protein network is associated with pathology of this disease. Thus, there is a requirement for systematic analysis of neuronal proteome from different regions of brain. A study by McKetney et al. has tried to address this by developing a proteomic atlas of nine anatomical regions of brain from three aged individuals [19]. Such atlases are helpful for understanding region specific neurodegeneration of brain.

Apart from brain tissues, protein aggregates such as extracellular amyloid-beta plaques and tau neurofibrillary tangles have also been extensively studied using proteomics techniques providing insights into their role in AD pathology and progression [20]. Liao et al. used laser-capture microdissection (LCM) coupled with LC-MS/MS analysis of frozen brain sections to report deep proteome of amyloid plaques for the first time identifying 488 proteins including multiple phosphorylation sites on the neurofilament intermediate chain [21]. Bai et al. performed a deep proteome analysis of detergent-insoluble protein aggregates from brain tissue and identified ~4000 proteins, including U1–70K and other U1 small nuclear ribonucleoprotein (U1 snRNP) spliceosome components in AD as well as MCI samples [22]. Such insights underscore the need for further functional studies to probe the exact role of aggregate-associated proteins in AD, and mass-spectrometry-based proteomics may prove to be an invaluable tool.

#### 2.1.2. Ageing Model Systems and AD Pathogenesis

The elucidation of molecular mechanisms and cellular signaling pathways, that are responsible for neuronal cell death and advancement of the disease, is critical for discovering novel therapeutic targets. There are several limitations to use human postmortem brain tissue for research purposes: major limitations being the availability of tissue, quality of the tissue, genetic heterogeneity, pre-terminal medication or concomitant disease, cause of death, and postmortem interval. This large variability in sample procurement and other confounding factors produce inconsistent results in proteomics analyses. In vivo animal model systems serve as a better alternative to postmortem brain tissue in understanding disease mechanism as many of these issues related to consistency can be controlled. Such models can also be used for studying the molecular progression of the disease as well as different region-specific changes in the brain. Multiple mouse models have been employed for studying AD-related neurodegeneration. Tauopathy mouse model with human P301L mutation along with non-transgenic littermate controls has been used by Vega et al. for two-dimensional gel electrophoresis (2-DiGE)-based proteomics. This proteome analysis identified alteration in Ezrin expression in the early stages of neurodegeneration in tauopathy models [23]. Mazi et al. identified early molecular events in AD pathogenesis by protein expression profiling of cortex, hippocampus, and cerebellum of transgenic mice carrying five familial AD mutations (5XFAD) at neonatal day 1 along with non-transgenic age-matched littermates [24]. In another study, alterations in O-GlcNAcyl-proteome in the brain of 12-month-old 3 × Tg-AD mice were compared with age-matched non-Tg mice. Lower levels of O-GlcNAcylation were identified in multiple proteins involved in AD progression [25]. Völgyi et al. studied the proteome of the mitochondria-associated ER membrane (MAM) isolated from the cerebral cortex of 3-months-old APP/PS1 mouse model of AD and age-matched C57BL/6 control mice. Mass-spectrometric analysis of these samples identified several altered proteins showing a strong relationship between the detected MAM protein changes and AD [26]. Hippocampus being an early region affected in AD, Li et al. carried out proteome profiling of hippocampus of APP_Sw,Ind_ J20 mouse along with control. They reported altered mTOR signaling and β-spectrin, Rab3-mediated APP trafficking and proteolysis as early molecular events associated with AD pathogenesis [27]. Systematic analysis of proteome, phosphoproteome, and sialylated N-linked glycosylation of APPswe/PS1ΔE9 mouse olfactory bulb, hippocampus, neocortex, and the brainstem revealed alteration in synaptic functions associated with dendritic spine morphology, neurite outgrowth, long-term potentiation, CREB signaling, and cytoskeletal dynamics [28]. Although use of such animal models has furthered our understanding of the disease, one major limitation of such models is that they fail to fully recapitulate disease symptoms.

#### 2.1.3. Formalin-Fixed, Paraffin-Embedded (FFPE)

Studies on animal models of AD have greatly improved our understanding of disease pathogenesis. However, translational clinical research is still heavily reliant on human tissue specimens. One of the other alternatives to fresh human tissue is formalin-fixed and paraffin-embedded (FFPE) tissue sample. These FFPE tissues can be stored for the long term and also have a plethora of associated clinical data like survival time and therapy response. Although such archives are immensely valuable, the use of formalin for fixation induces cross-linkages in proteins and makes it technically challenging for proteomic analyses. Several researchers have successfully adopted proteomics protocols for analyses of FFPE samples, and recent technological advancements have yielded reliable recovery and reproducible quantification of proteins comparable to fresh frozen tissues. Drummond et al. isolated neuronal cells from temporal cortex FFPE samples with severe Alzheimer’s using the LCM-LC-MS/MS technique and identified more than 400 proteins of which 78% were of neuronal origin and about 200 proteins were associated with Alzheimer’s disease [29]. Similarly, another study on amyloid plaques and NFTs micro-dissected from FFPE tissue identified about 900 proteins in plaques and ~500 proteins in NFTs [30]. These studies demonstrate the viability of using proteomics for such archival specimens akin to fresh frozen tissue samples for the understanding of disease pathogenesis. However, further challenges that are yet to be reliably addressed include limiting the effects of formaldehyde-induced protein modification on protein recovery as well as investigating the repertoire of formaldehyde-induced protein modifications that may affect protein identification in shotgun proteomics [31]. Another relevant area that is still largely unexplored in the context of Alzheimer’s disease is the use of FFPE samples for the identification of changes in the post-translation modifications.

### 2.2. Proteomics and Fluid Biomarkers Discovery

The goal of the protein marker discovery is to detect a protein or panel of proteins that differentiates affected patients with a specific disease from healthy individuals. For biomarker analysis, it is preferred that it would be accessible through minimal invasion. There are multiple studies on identifying markers for AD using biofluids such as cerebrospinal fluid [32], plasma [33], saliva [34], and urine [35].

#### 2.2.1. Cerebrospinal Fluid

Cerebrospinal fluid (CSF) is a clear liquid produced in the ventricles of the brain that surrounds and protects the central nervous system, making it an important source of biomarkers for AD. As CSF surrounds the brain tissue, it would reflect overall brain physiology and processes like synapses loss and neuronal damage [36]. It has been hypothesized that changes in the composition of CSF proteome may reflect the CNS-associated idiosyncrasies in protein expression that are complementary with neurodegenerative disorders [37]. Several studies have employed a discovery proteomics approach for the detection of new AD biomarkers in CSF [38,39]. Multiple early studies have used 2-D protein electrophoresis for the analysis of AD and control CSF samples. These studies identified differential proteins and potential AD-CSF markers. A major limitation of such studies was their limited coverage of the proteome [40,41,42]. There are several studies published on deep proteomic analysis of CSF using depletion of high-abundant proteins coupled with high-resolution mass spectrometry to increase coverage of low-abundant proteins [32,43]. Previously known AD markers including Tau and amyloid-beta peptides have been identified across multiple mass-spectrometry-based proteomics studies and have been successfully developed into targeted assays for quantitation [44,45,46,47]. Multiple studies by Barthelemey et al. and groups have studied tau phosphorylation in CSF, brain, and plasma and reported an association between site-specific changes in tau phosphorylation and evolution of stages of dominantly inherited Alzheimer’s disease [48,49,50]. Another large-scale analysis of the CSF from AD patients has identified a consistent glycolytic signature [51]. From such discovery proteomics analyses, as well as from other molecular analyses, several proteins associated with AD pathogenesis have been identified. These proteins have been taken forward for validation of potential biomarkers on a large number of samples using a targeted proteomics approach [52,53]. Attempts have also been made toward the identification of secretary post-translationally modified proteins in CSF as markers for AD [54]. Further, a meta-analysis of CSF proteomics studies by Pedrero-Prieto et al. identified a panel of 27 proteins and 21 peptides highly altered in AD with consistent expression at least across three studies [55]. Although CSF-biomarker studies have identified several novel protein markers for AD, these studies are largely constrained by limited sample size. The findings of these studies now need to be taken to the next level and validated on multiple large independent cohorts across different stages of the disease to realize their full potential.

#### 2.2.2. Serum

Serum or plasma represents the largest version of the human proteome present in any sample as it also contains proteins from all tissue as leakage markers apart from classical “plasma proteins” [56,57]. Serum-based proteomic biomarkers make for a more practical approach for the implementation as they are minimally invasive and inexpensive. At the same time, measuring proteins in serum is technically challenging because of its complex composition, a large dynamic range of protein abundance spanning 12 orders of magnitude, and dominating abundance of albumin [56]. As 99% serum proteome is composed of albumin, evaluating changes in the remaining small fraction of proteins based on disease is an uphill task for the most advanced mass spectrometers as well. One widely used strategy to access the low-abundant proteome of plasma is depletion of these high abundant proteins using an antibody-based approach [58,59,60,61]. Another major hurdle is the blood–brain barrier, because proteins detected in blood and their relation to the brain is uncertain. However, with aging and in neurodegenerative disease, the blood–brain barrier is disturbed which results in increased permeability, and there is a chance of detecting disease-specific proteins in the blood [62].

Several discovery proteomics [63,64,65] as well as targeted proteomics studies were carried out for the identification of serum protein markers [66,67]. A recent review by Zetterberg et al. has curated a timeline for recent biomarker studies aimed at development of clinically implementable blood test for Alzheimer’s summarizing different technologies employed for such research [68]. Dayon et al. used proteomics workflow on plasma samples from 120 individuals and identified panels of proteins that improve the diagnostic accuracy of CSF-defined AD pathology and amyloidosis [69]. Dey et al. used extensive pre-fractionation to circumvent the dynamic range problem and carried out deep proteomic profiling of undepleted human serum proteome for the biomarker discovery for Alzheimer’s disease identifying more than 4000 proteins. They reported a panel of 30 proteins significantly altered between control and Alzheimer’s samples most of which were related to mitochondrial function and further validated downregulation of AK2 and PCK2 using multiplexed-targeted proteomics strategy [33]. Another study from the same group integrated data from the brain, CSF, and plasma across multiple datasets. They showed that changes in mitochondrial protein as the most consistent AD signature carried over from brain cortex to CSF and serum [14]. Using an enrichment strategy combined with targeted MS Barthelemy et al. measured attomolar concentration of tau isoforms in plasma and reported that p-tau-217 is more accurate than p-tau-181 for detecting abnormal CNS tau metabolism [70]. Recently, Nakamura et al. developed an immunoprecipitation (IP) and MALDI-MS-based approach to measure plasma Aβ and proposed an AD composite biomarker based on (APP)_669–711_/Aβ_1–42_ and Aβ_1–40_/Aβ_1–42_ ratios [71]. Han et al. used selected reaction monitoring for quantification of the apolipoprotein E (ApoE) in the serum of the AD patients. They identified a significant decrease in serum ApoE levels in AD patients compared to controls [66]. These studies, although limited by sample size, demonstrate serum biomarkers as a viable option despite several challenges posed by being a complex matrix.

#### 2.2.3. Urine

Urine is formed through the filtration of plasma by glomeruli in the kidneys. Urine contains water, glucose, salt, and other metabolites and proteins derived from the serum proteins. Thus, urine can provide readout of the systemic physiology [72]. Increased g levels of urinary Aβ42 have been demonstrated in AD patient samples and have been proposed as a marker for both AD diagnosis and monitoring [73]. Watanabe et al. identified 109 proteins that significantly differed between AD patients and controls from 18 AD and control urine samples [74]. In a similar study by Yao et al., urine samples from AD patients and healthy controls were analyzed using iTRAQ-based quantitative proteomics. They identified SPP1, GSN, and IGFBP7 as potential urine protein biomarkers for AD [35]. These studies demonstrate the feasibility of using urine as a target fluid for biomarker discovery. Although urine would turn out to be a popular biofluid for the biomarker analysis, further efforts in this direction are required for the identification and validation of robust candidates.

#### 2.2.4. Saliva

Saliva is a physiological fluid that contains mucous and serous secretions containing mucin, alpha-amylase, and other proteins. For biomarker analysis, CSF is a relatively invasive procedure that requires the participation of specially trained medical professionals. This makes CSF a non-ideal sample for screening and early diagnosis. Saliva is noninvasive and comparatively very easily accessible compared to both blood and CSF. Expression of tau mRNA and amyloid-β precursor protein (AβPP) has been reported in the salivary gland and human salivary epithelial cells, respectively [75,76]. Min Shi et al. have also reported the identification of salivary tau and p-tau as AD biomarkers by immunoprecipitation and mass-spectrometry-based identification [34]. Although studies demonstrating the use of saliva proteomics for AD biomarkers detection are limited, such preliminary reports suggest that salivary tau species could be ideal biomarkers for AD diagnosis especially for the early stages of the disease. Such markers also have the potential to be used for screening asymptomatic subjects, dramatically increasing the window for therapeutic interventions.

#### 2.2.5. Ocular Biofluid

Currently, an autopsy is the only method that gives a confirmed AD diagnosis. The eye shares a neural and vascular resemblance to the brain, thus providing an opportunity to access the cerebral pathology [77]. Visual symptoms have been reported in many AD patients, and this has piqued an increased interest in discovering ocular biomarkers that might be related to the pathology [78,79]. Several proteomics studies have been carried analyzing the tear proteome to identify AD markers [80,81]. Proteomic analysis of the retinal tissue and vitreous humor fluid from glaucoma patients shows alternation of the several proteins involved in the pathophysiology of AD pointing toward defects in mitochondrial oxidative phosphorylation machinery [82]. These ocular proteomics studies open a new window for the identification of the potential biomarker for AD. In the future, systematic in-depth proteomics analysis of the eye and related structures and fluids in the context of the AD is needed.

## 3. Proteomics Technologies Employed for Understanding AD Pathogenesis and for Biomarker Discovery

**Figure 2:**
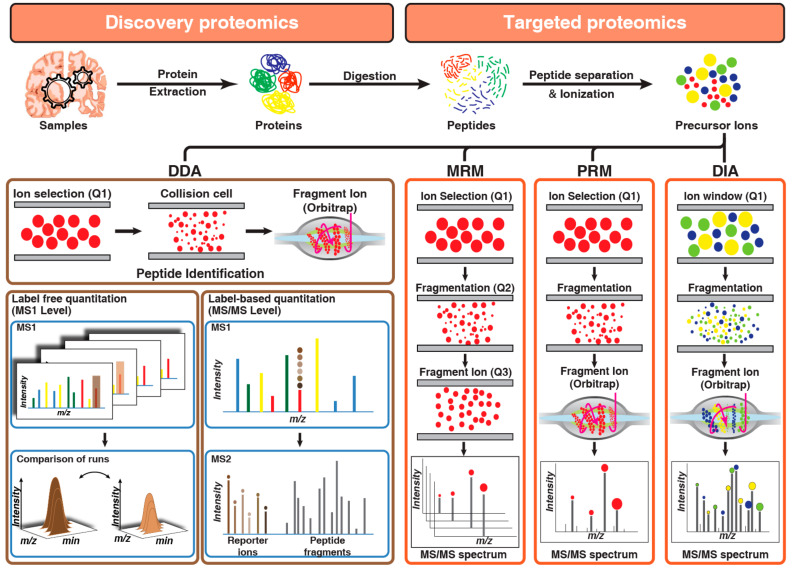
Overview of discovery and targeted proteomics technologies: Panel 1 describes label-free and label-based quantitation techniques used in discovery proteomics to identify differentially expressed proteins across samples by comparing either intensities and peak area of peptides (label-free quantitation) or reporter ion intensities (label-based quantitation) generated after fragmentation of labeled peptide precursors. Panel 2 describes targeted proteomics techniques used for monitoring peptides across samples by either sequential selection and monitoring of precursor-product ion pair (MRM) or simultaneous monitoring of all fragment ions of a selected precursor ion (PRM) or targeted extraction of fragment ion intensities from a mixed spectrum of fragment ions generated from multiple precursor ions selected by using a small window of m/z (DIA).

Evolution of the proteomics technologies started from the development of the separation of proteins by two-dimensional polyacrylamide gel electrophoresis [83]. Previously several studies have used 2-D gel electrophoresis for the understand pathogenesis of AD [63,84,85]. Subsequently, exponential development in the shotgun proteomics, driven by high-resolution mass spectrometers, has largely superseded two-dimensional (2D) gel-based proteomics both in terms of coverage or depth and throughput. Proteomics technologies that are employed toward the identification of the AD-related changes in CSF, plasma, and tissue are broadly categorized as discovery or targeted proteomics (Figure 2). The discovery-based approach has been primarily used for the quantitation of changes in molecular signaling networks that are associated with AD pathology. While a targeted proteomics approach is being used for monitoring and quantitation of proteins that are already known to be associated with AD pathogenesis and can be used as a candidate marker.

### 3.1. Discovery-Based Proteomics Analyses

Data-dependent acquisition or information-dependent acquisition has been the cornerstone of shotgun-discovery-based proteomics technology. In the data-dependent acquisition (DDA) in each duty cycle, the instrument cycles through first a short high-resolution MS1 survey scan of the peptides and based on the peptide intensity it selects potential precursor ions for fragmentation followed by series of quick low-resolution MS2 scan for detection of the fragment ions (product ions). The resolution setting and automatic-gain control for MS1 and MS2 scans are optimized to get fast scan speed and lower duty cycle while providing high accuracy for precursor identification. Extensive pre-fractionation strategies or enrichment strategies are employed to either increase the proteome coverage or to access low abundant post-translationally modified proteome. Data from DDA is analyzed by comparing the experimental mass spectrum to a theoretical mass-spectrum generated from a protein reference database or a spectral library matching using different vendor-specific or open-source algorithms. Further, in discovery-based analysis, two often-used proteomics approaches are label-free and isobaric multiplex labeling strategies for relative quantitative proteomics

#### 3.1.1. Label-Free Quantitative Proteomics

Label-free quantitative (LFQ) proteomics approaches take advantage of the correlation between high-resolution LC/MS extracted ion currents (XIC) and peptide abundances for the identification of differentially abundant proteins between the different sample groups [86]. Signals obtained from XICs with identical retention times and m/z values can be directly compared to measure statistically significant differences between sample groups. A quantitative label-free proteomics approach has been used for the assessment of technical variability related to sample processing, instrument conditions, and detection of inter-individual variation in cerebrospinal fluid biomarker analysis [87]. Label-free proteomic analysis approach has also been used for quantitation of proteins for Alzheimer’s disease (AD) brains versus normally aged brains [88] and membrane-enriched proteome from postmortem human brain tissue in Alzheimer’s disease [89]. Another study compared the label-free quantitative proteomic analysis of cerebrospinal fluid glycoproteins and endogenous peptides in subjects with Alzheimer’s disease, mild cognitive impairment, and healthy individuals [90]. Although LFQ strategies provide an advantage in terms of ease of application, throughput and proteome coverage presents a formidable challenge.

#### 3.1.2. Isobaric Multiplex Labeling Strategies for Relative Quantitative Proteomics

Isobaric multiplex labeling strategies allows for relative quantitation of proteins across multiple samples in a single LC-MS run, thereby greatly increasing the throughput. Digested peptides from each sample are N-terminally tagged by one of the isobaric labels either with isobaric tag for relative and absolute quantitation (iTRAQ) or tandem mass tag (TMT). With iTRAQ reagent, eight samples can be multiplexed, while TMT reagent allows multiplexing up to 16 samples. After complete labeling of the peptides, samples are pooled followed by fractionation and LC-MS/MS analysis which drastically improves the proteome coverage. High-resolution tandem mass spectrometry can easily distinguish these tags where the relative intensity of these tags corresponds to the relative abundance of the peptides across the samples.

Several studies have carried out proteomics analysis for the discovery of biomarkers using either iTRAQ or TMT. Sathe et al. used TMT-based multiplexing for the identification of CSF-based biomarker for Alzheimer’s disease. In this study, they identified several known and novel protein markers for AD. In this study, several previously known AD markers including MAPT, NPTX2, SCG2, VGF, GFAP, SST, and NCAM1 as well as novel biomarkers such as GSN, PKM, and YWHAG were identified [32]. In another study, brain tissue from AD, AsymAD, and controls was analyzed using TMT-based quantitative mass spectrometry. They identified the alteration of 350 proteins between AsymAD and AD [12]. Adav et al. used iTRAQ-based quantitative proteomics for the identification of alterations in mitochondrial proteome of the human brain tissues of healthy and AD individuals. They identified the de-regulation of the electron chain complex and ATP-synthase as the major driver of AD pathology [91]. Multiplexing of the samples using isobaric labeling revolutionized the field of quantitative proteomics. So far, many large-scale proteomics analysis in AD is driven by the advancement of isobaric labeling strategies [15,16,17,92].

#### 3.1.3. Post-Translational Modification Proteomics

Post-translational modifications (PTMs) are key regulators of cell signaling. There are more than 200 types of PTMs that are known to control cellular functions. Phosphorylation of proteins, at serine, threonine, or tyrosine residues, are major post-translational modifications that control cellular signaling. Protein phosphorylation and dephosphorylation is a reversible process that is regulated by kinases and phosphatases. Global characterization of changes in protein phosphorylation is needed for the understanding of the complex regulatory circuits needed for cellular response to a stimulus.

Quantitative phosphoproteomics analysis of the hippocampus of the Alzheimer’s disease subjects by Domenico et al. identified significant alteration in the phosphorylation of the proteins involved in important neuronal processes such as formation, outgrowth, and guidance of neurites [93]. In another study, phosphoproteomic analysis of cerebral cortex carried out on an early onset mouse model (TgCRND8) of Alzheimer’s disease by Chou et al. identified alteration in the neuronal and glial signaling pathways [94]. Dammer et al. used the IMAC-based method for the enrichment of the phosphopeptides from the frontal cortex of the AD subjects and control individuals, identifying 142 hyperphosphorylated proteins in AD samples. Differential phosphorylation of heat shock protein 27 (HSPB1) and crystallin-alpha-B (CRYAB) was further validated by Western blotting [95]. Sathe et al. used TMT multiplexing along with an IMAC-based phosphoproteomics approach for the identification of alterations in phosphoproteome of the frontal gyrus of AD brains [96]. Chen et al. carried out a phosphoproteomic analysis of selenate-treated Alzheimer’s disease model cells N2aSW. They used 2D gel electrophoresis and Pro-Q diamond-based phosphoproteins staining leading to the identification of 65 differentially stained phosphoproteins spots after mass-spectrometric analysis. These altered phosphoproteins were involved in multiple neuronal processes including oxidative stress, cysteine and methionine metabolism, and energy metabolism [97]. Triplett et al. also used a similar approach for phosphoprotein profiling of inferior parietal lobule of subjects with AD, MCI, PCAD, and control brain. This reported significant changes in the phosphorylation of proteins involved in energy metabolism, neuronal plasticity, signal transduction, and oxidative stress response [98]. Kempf et al. carried out proteomics, phosphoproteomics, and N-glycosylation changes in the four different part of the brain of the APP/PS1 Alzheimer’s mouse model along with a wild-type mouse. These analyses identified alteration of brain-region specific response in CREB-mediated synaptic signaling with APP/PS1 mutation [99]. In neuron’s, abnormal changes in Tau protein, such as phosphorylation and aggregation, are considered hallmarks of Alzheimer’s disease. Phosphoproteomics analysis of the postmortem human brain tissues revealed that among the differentially phosphorylated sites, approximately 21% of significantly altered phosphopeptides in AD tissue were derived from tau [95]. Using cell- and animal-based-bioactivity assay along with proteomics Dujardin et al. reported patient-to-patient heterogeneity in the hyperphosphorylated species of soluble, oligomeric, seed-competent tau [100]. Chou et al. has discussed the effects of multiple post-translational modifications including phosphorylation, acetylation, SUMOylation, O-GlcNAcylation, and ubiquitination of tau on AD pathology in their review article [101]. A recent review by Rayaprolu et al. describes how a network-based proteomics approach can be leveraged to improve our narrow understanding of AD biology beyond amyloid plaques and tau phosphorylation [102]. These post-translation modification-related proteomics studies on human postmortem brain specimen or animal models are useful for the understanding of the altered signaling networks in AD pathogenesis. One of the major advantages of phosphoproteomics studies is that the altered kinases identified by them will be also helpful as potential therapeutic targets in future.

### 3.2. Targeted Proteomics Analyses

Once the discovery data are acquired, rigorous statistical methods are applied to identify significantly differential proteins that can be used as potential markers for the disease. Multiple tools including Perseus [103], PANDA [104], and DAnTE [105] are available for such statistical analysis. To quantitate the abundance of these markers reproducibly, highly robust and reproducible methods are required. Although discovery-based data-dependent shotgun proteomics is routinely used for biomarker identification, the major limitation of this approach is its irreproducibility and imprecision. If numerous peptide species co-elute and appear in a single MS1 scan, then DDA stochastically samples only the most abundant peptides and misses the rest. Another major limitation of DDA is that it intentionally samples each peptide species only once or twice to increase the coverage, preventing precise absolute quantification that requires multiple measurements per peptide. To overcome this limitation, targeted proteomics approaches are used that provide reproducible quantitation. In general proteomics, workflow DDA and targeted proteomics are used in tandem or parallel. In targeted proteomics, multiple- or parallel-reaction monitoring (MRM or PRM) and data-independent analysis (DIA) are generally used (Figure 2).

#### 3.2.1. Multiple-Reaction Monitoring

In a targeted proteomic analysis, proteotypic peptides are selected as a proxy for quantifying proteins of interest. Single-reaction monitoring (SRM) or multiple-reaction monitoring (MRM) analyses are routinely used for quantitation of proteins and require a tandem mass spectrometer with more than one mass analyzer. In both SRM and MRM experiments, the first mass analyzer, quadrupole (Q1), selects the precursor ion and the third quadrupole (Q3) selects the desired fragment ion using narrow mass windows. In the second quadrupole (Q2) or collision cell, precursor ions are fragmented via collisional induced disassociation (CID). Therefore, SRM requires a signal from both precursor and fragments ion pair to generate a positive result. This allows SRM to achieve high specificity along with very low background noise thereby enhancing the sensitivity of detection. Monitoring multiple precursor-fragment ion transitions for the same or different analytes in a single mass-spectrometer analysis is referred to as multiple-reaction monitoring (MRM). Several studies have employed an MRM-based targeted proteomics approach for biomarker analysis for Alzheimer’s disease.

Multiple-reaction monitoring used for the quantification of Alpha-, Beta-, and Gamma-synuclein identified an increase in concentrations in the cerebrospinal fluid of Alzheimer’s patients [106]. Similarly, Wesseling et al. reported heterogeneity on tau phosphorylation sites across AD patients using FLEXItau, an MRM-based assay, and identified multiple features in tau protein that can be targeted for disease intervention [107,108]. Paterson et al. developed a multiplex MRM assay for the assessment of 54 candidate biomarkers in the cerebrospinal fluid (CSF). They identified alteration in proteins involved in glucose metabolism and neuroinflammation in AD patients [109]. Wildsmith et al. developed an MRM assay for the absolute quantitation of 30 candidate biomarkers in longitudinal CSF samples collected from aged, cognitively normal control. These markers were compared with already known markers including CSF Aβ42, tau, and p-tau181. Four CSF markers including amyloid precursor protein, neuronal pentraxin receptor, NRCAM, and Chromogranin A correlated with a significant longitudinal change in AD [110]. Synaptic degeneration is a major hallmark for AD. Chang et al. developed a synapse, enrichment and label-free, MRM-MS method for the quantitation of synaptic proteins in human brain samples. They demonstrated the quantitative accuracy of the method and observed significant alteration in the synaptic proteins [111]. In another study, Chang et al. monitored a panel of 10 synaptic proteins in AD brain samples using label-free MRM-MS. They reported significantly higher levels of peroxiredoxin-1 and energy metabolism-related enzymes *viz.* creatine kinase B and fructose-bisphosphate aldolase C in AD hippocampus tissue [112]. Although ApoE4 is the most important genetic risk factor for AD, differential levels of total blood ApoE have been reported between carriers of different ApoE genotypes [113,114,115]. Several groups reported successful identification of ApoE isoforms in blood by quantifying allele-specific peptides with MRM-MS in complete concordance with classical genotyping [113,116]. Such studies demonstrate the value of the targeted mass spectrometry-based approach in biomarker discovery.

#### 3.2.2. Parallel-Reaction Monitoring

Parallel-reaction monitoring is a rapidly emerging application of high-resolution mass spectrometry for targeted proteomics analysis. In parallel-reaction monitoring, the third quadrupole of a triple quadrupole mass spectrometer is replaced with a high resolution and accurate mass analyzer to allow parallel detection of all target product ions in one concerted high-resolution mass spectrum [117]. PRM analysis has several advantages over MRM for targeted proteomics applications. PRM provides better specificity as all potential fragment ions of a peptide precursor ion are recorded in PRM, instead of just 3–5 transitions recorded in an MRM experiment [118]. Furthermore, PRM has a higher tolerance for co-isolated background peptides as numerous ions are available for identification and quantitation purposes and the presence of interfering ions in a full mass spectrum is less disruptive to overall spectral quality [119]. In addition, setting a PRM assay does not require prior knowledge and pre-selection of target transitions before analysis as all product ions are recorded. We have used label-free PRM analysis of identified AD-CSF candidate from discovery analysis. We demonstrated that NPTX2, in combination with PKM or YWHAG in CSF differentiate AD from control samples [32]. In another study, Brinkmalm et al. also used PRM-MS for quantification of neurosecretory proteins including Secretogranin-2, neurosecretory protein VGF and chromogranin A among others as AD marker panel [120]. Using PRM-MS, Öhrfelt et al. detected a significant increase in the CSF levels of synaptotagmin-1 in patients with Alzheimer’s associated dementia [121]. PRM-MS was also used for quantification of several proteins involved in vesicular transport and synaptic stability by Duits et al. They detected a significant increase in patients with MCI, especially in patients with MCI progressing to AD dementia [122]. Barthélemy et al. used PRM-MS to quantify the abundance of 29 unique phosphorylation sites in tau proteins in the brain and 12 sites on truncated tau in CSF in humans with and without AD. They reported that the abundance of phosphorylated sites varied in a site-specific manner between CSF and brain and these sites are differentially affected by Alzheimer’s disease [48]. These targeted proteomics assays are popular because of their high specificity and multiplexing abilities and have become a very valuable tool for assessing proteins for which commercial antibodies are not available.

#### 3.2.3. Data-Independent Analysis

Data-independent analysis (DIA) or sequential window acquisition of all theoretical mass spectra [123] is a recent advancement and a rapidly growing technology in proteomics analysis. DIA experiments are conducted on a hybrid instrument with full scan data being recorded in high-resolution mass analyzers such as time-of-flight (ToF) or Orbitrap [124]. Similar to DDA, in DIA the instrument cycles through an MS1 scan followed by a series of MS2 scans. However, instead of selecting a single precursor ion based on intensity for MS2, in DIA analysis, all precursor within a small defined mass-to-charge (m/z) window are subjected to MS2 fragmentation, and high-resolution fragment ion mass spectra are recorded for all the peptides within the window. This mass window is then stepped across the entire mass range in an overlapping manner, systematically recording MS/MS data from all detectable precursor ions present in a biological sample generating a complete and permanent digital archive of the sample. DIA thus provides advantages of both the DDA (high throughput) analysis as well as parallel reaction monitoring (high reproducibility and consistency) while circumventing their limitations. In DIA methods, as all precursors are recorded, it overcomes the limitation of precursor abundance-driven biased fragmentation nature of DDA. DIA methods also score over MRM/PRM methods as these methods are limited by the number of targets that can be monitored in a single MS run.

However, the major challenges of DIA-MS lie in data analysis and interpretation of the mixed tandem spectra originating from multiple precursors. For the analysis of the DIA data, several tools were available including Skyline [125], Spectronaut (Commercial), and OpenSWATH [126]. In DIA data analysis, the spectral library-based search method is commonly used for the identification of precursor peptides primarily using a proteome library generated using DDA analysis. A major limitation of this approach is the time taken and the sample consumption for library generation. Advancements in the field now allow a spectral-library independent analysis using algorithms such as DIA-Umpire [127] that combines the use of conventional database searching and protein-inference tools. Deep-learning architectures such as Prosit can learn and predict both the chromatographic retention time and the fragment ion intensity of any peptide with extremely high quality [128]. These methods provide alternative solutions to data analysis and interpretation bottlenecks in the DIA proteomics approach.

Chang et al. used DIA for quantifying the synaptic proteome of synaptosomes isolated from the hippocampus and motor cortex tissue from Alzheimer’s disease cases and subjects with normal aging. They identified a total of 2077 unique proteins and reported differential expression of 30 protein including 17 novel proteins in AD [129]. Bader et al. characterized systems-wide changes of CSF protein levels that accompany AD in three independent cohorts (197 individuals) consistently quantifying more than 1000 proteins from a few microliters of samples [51]. They reported 20 proteins to be differentially expressed in AD CSF samples including tau, superoxide dismutase 1, PARK7, YKL-40, and novel biomarker candidates such as YWHAG, PKM, and ALDOC. Bader et al. provide a novel application of DIA proteomics strategies to drive a shift in the clinical proteome paradigm toward a population-wide discovery and validation of biomarker candidates that exonerate the individual-specific effects observed in small pilot studies.

## 4. Conclusions and Future Directions

Recent developments in sample preparation methodologies along with high-throughput mass spectrometry have improved the proteome coverage. Broadly, the proteomic analysis methods can be divided into discovery and targeted mass-spectrometry-based methods. Several quantitative proteomics methods including label-free, isobaric labelling based in the discovery platform and MRM, PRM and DIA in the targeted platform have been employed for AD biomarker discover and understanding AD pathogenesis. These advancements in technologies have rapidly generated several datasets on various biofluids, tissues, and animal models related to Alzheimer’s disease. In this review, we have discussed these AD-related studies on the various specimens as well as on different mass-spectrometry platforms in detail. Although these studies have dramatically improved our understanding of disease pathology, as the complexity of the disease is high, studies at a large scale in diverse populations will be required in future for the identification of the potential biomarkers. One of the other limitations in the identification of robust and reliable biomarker candidates is the current “triangular strategy” of discovering candidates from a smaller subset of samples and validation in a larger cohort. Where many of the identified candidates turn out to be specific to the discovery cohort and fail at validation stages in larger cohorts. Such limitations are now being addressed by a shift in the study design of clinical discovery proteomics with a “rectangular strategy”. Such strategies employ high proteome depth workflows for simultaneous discovery and validation on hundreds of samples in a population-wide setting. In the future, it is also necessary to have a stringent reporting system for study and patient selection criteria, clinical metadata, and parameter used for analysis. This information will be useful for comparison of the datasets as well as to build a knowledge-base that will help to further our understanding of the disease and associated neurodegeneration.
